# Overcoming segmentation bottlenecks in brain tissue for spatial proteomic inference at population scale

**DOI:** 10.64898/2026.10.22.761199

**Published:** 2026-10-24

**Authors:** Liang You, Jamie L. Durbin, Guanqun Meng, Wayne Stallaert, Guang Li, Shikhar Uttam, Hao Feng, Jill R. Glausier, Jiebiao Wang

## Abstract

In spatial proteomics across large cohorts, downstream estimates from tens of thousands of images depend on accurate cell segmentation. Yet pretrained segmentation models struggle with morphologically complex cell bodies in human brain tissue, and how domain adaptation affects downstream discovery remains unclear. Here, we introduce SPInfer, a workflow for spatial proteomic inference developed using 5,832 manually annotated cell bodies from 120 immunofluorescence images of the human prefrontal cortex. After benchmarking eight segmentation methods spanning classical, convolutional, and foundation models, we adapted Cellpose-SAM, raising cell segmentation F1 from 0.37 to 0.84 and recovering cell compositions concordant with matched single-nucleus RNA-seq and spatial transcriptomics. Across 14,765 images from 69 postmortem donors, identical Alzheimer’s disease pathology models found no significant associations after false discovery rate correction using zero-shot masks, versus four using fine-tuned masks; three linked higher OLIG2-positive fractions to greater neurofibrillary tangle and plaque burden. Using the fine-tuned masks, a graph attention network over nuclear neighborhoods classified four cell types without marker information, and differential expression analysis linked cell morphology to gene expression. This provides a pipeline for fine-tuning foundation models to segment complex tissues, enabling biological discovery in spatial proteomics at the population scale.

